# Supplementary material for: Measuring the Benefits of Mass Vaccination Programs in the United States
Source: Vaccines (Basel). 2020 Sep 29;8(4):561. doi: 10.3390/vaccines8040561 (PMC7712487; doi:10.3390/vaccines8040561)
Supplement: Supplementary file 1 [file vaccines-08-00561-s001.pdf]

Measuring the Benefits of Mass Vaccination Programs in the United States: Online Supplement

Tables and Figures

Table S1. Basis for figures concerning measles
- A. Number of measles cases, 1959-1962
- B. Estimated number of permanent disabilities from measles for 2014 in the absence of mass vaccination
- C. Percentage of severe measles cases that have low levels of vitamin A

Figure S1. Decline in measles mortality, 1900-1960
Table S2. Basis for figures concerning mumps
- A. Number of mumps cases, 1963-1966
- B. Estimated number of permanent disabilities from mumps for 2014 in the absence of mass vaccination

Table S3. Basis for figures concerning rubella
- A. Number of rubella cases, 1960-1968
- B. Estimated number of cases of CRS, 1960-1968

Table S4. Basis for figures concerning tetanus
- A. Estimated incidence of tetanus, 1943-1945
- B. Tetanus case fatality rate among unvaccinated individuals <80 years of age, 2000-2008

Figure S2. Decline in tetanus mortality, 1900-1945
Table S5. Basis for figures concerning diphtheria
- Estimated number of deaths from diphtheria for 2014 in the absence of mass vaccination

Figure S3. Decline in diphtheria mortality, 1879-1945
Table S6. Basis for figures concerning pertussis
- A. Estimated number of cases of pertussis for 2014 in the absence of mass vaccination
- B. Pertussis reported case fatality rate among unvaccinated infants <3 months of age, 2012-2014
- C. Estimated number of cases of pertussis among infants <3 months of age for 2014 in the absence of mass vaccination
- D. Number of pertussis deaths and percentage that occurred among infants <3 months of age, 1943-1945

Figure S4. Decline in pertussis mortality, 1900-1945
Table S7. Basis for figures concerning polio
- A. Estimated number of cases of paralytic poliomyelitis, 1935-1954
- B. Estimated number of poliomyelitis cases resulting in permanent disability or death, 1935-1954
- C. Estimated case fatality rate of paralytic poliomyelitis, 1935-1954
- D. Estimated number of poliomyelitis cases resulting in death or permanent disability for 2014 in the absence of mass vaccination
- E. Estimated risk of permanent disability or death from poliomyelitis in children <10 years of age based on tonsillectomy and rest status, 1935-1954
- F. Estimated risk of permanent disability or death from poliomyelitis in individuals 10-39 years of age based on tonsillectomy and rest status, 1935-1954
- G. Percentage of population that had no tonsils, 1935-1954
- H. Percentage of poliomyelitis cases that rested after significant onset of symptoms by type of paralysis and severity, 1935-1954

Table S8. Basis for figures concerning *Haemophilus influenzae* type b
- A. Estimated number of cases of invasive Hib in the absence of mass vaccination, 1994-2000
- B. Invasive *H. influenzae* tracking in children <5 years of age, 1994-2000

C. Estimated number of cases of invasive Hib resulting in death or permanent disability in the absence of mass vaccination, 1994-2000

D. Protective effect of breastfeeding against invasive Hib measured in 1997 Swedish study of children <6 years of age

E. Estimated number of cases of invasive Hib resulting in death or permanent disability for 2014 in the absence of mass vaccination based on breastfeeding status

F. Estimated risk of invasive Hib based on breastfeeding status in the absence of mass vaccination, 1994-2000

Table S9. Basis for figures concerning hepatitis B

A. Estimated number of cases of hepatitis B for 2014 in the absence of mass vaccination

B. Estimated number of cases of fatal fulminant hepatitis B for 2014 in the absence of mass vaccination

C. Estimated number of hepatitis B cases leading to death from chronic infection before age 80 for 2014 in the absence of mass vaccination among various age groups

D. Estimated number of hepatitis B cases leading to death before age 80 for 2014 in the absence of mass vaccination among adults and adolescents at high risk of exposure

E. Estimated number of hepatitis B cases leading to death before age 80 for 2014 in the absence of mass vaccination among children at high risk of exposure

Table S10. Basis for figures concerning varicella

Number of varicella cases, 1991-1994

Table S11. Basis for figures concerning human papillomavirus

A. Estimated number of HPV cases leading to death before age 80 for 2014 among women

B. Estimated number of HPV cases leading to death before age 80 for 2014 among men

Table S1. Basis for figures concerning measles.

**A. Number of measles cases, 1959-1962**<br><br>Before licensure of the measles vaccine, there were an annual 400,000 reported cases of measles [1]. However, similar to varicella (Table S10), because almost everyone in the population contracted measles before adulthood, the number of measles cases was actually an average of one birth cohort size (4 million cases) [1]. Consequently, reported cases comprised only 10% of all cases, and the case fatality rate based on reported cases of measles (1 in 1,000) [2] is greater than the case fatality rate with respect to all measles cases.
**B. Estimated number of permanent disabilities from measles for 2014 in the absence of mass vaccination**<br><br>Measles encephalitis occurs half as often as measles-related death, and 25% of measles encephalitis cases result in residual neurologic damage [3]. Therefore, based on an estimated 402 measles deaths for 2014, we calculated 201 (= 50% of 402) cases of measles encephalitis and 50 (≈ 25% of 201) measles cases resulting in residual neurologic damage. In addition, there are an estimated 6 to 22 cases of subacute sclerosing panencephalitis per million measles cases [4]. We used the midpoint of this range, 14 cases per million measles cases, to estimate 56 (14×4) cases of subacute sclerosing panencephalitis in the absence of mass vaccination.
**C. Percentage of severe measles cases that have low levels of vitamin A**<br><br>Studies of vitamin A levels in measles patients show that the percentage of measles cases with low vitamin A increases with disease severity [5]. A study of measles cases in New York, 36% of which were hospitalizations and none of which were fatal, found that 22% to 48% of all measles cases were low in vitamin A [6]. A study of measles cases in Wisconsin, 80% of which were hospitalizations and 2% of which were fatal, found that 72% of all measles cases were low in vitamin A [7]. A study of measles cases in Cape Town, South Africa, 100% of which were hospitalizations and 6% of which were fatal, found that 92% of all measles cases were low in vitamin A [5].<br><br>The 92% observed in the Cape Town study is consistent with the data collected in Wisconsin and New York. In the Wisconsin study, if 92% of the hospitalized cases were low in vitamin A, 73.6% (92% of 80%) of all measles cases would be expected to be low in vitamin A, similar to the actual 72% found in the study. In New York, if 92% of the hospitalized cases were low in vitamin A, 33% (92% of 36%) of all measles cases would be expected to be low in vitamin A, well within the range of 22% to 48% found in the study. Consequently, because we are considering the most severe cases of measles in this report, those resulting in permanent disability or death, we estimated that 92% of such cases have low levels of vitamin A.

Figure S1. Decline in measles mortality, 1900-1960 [8]

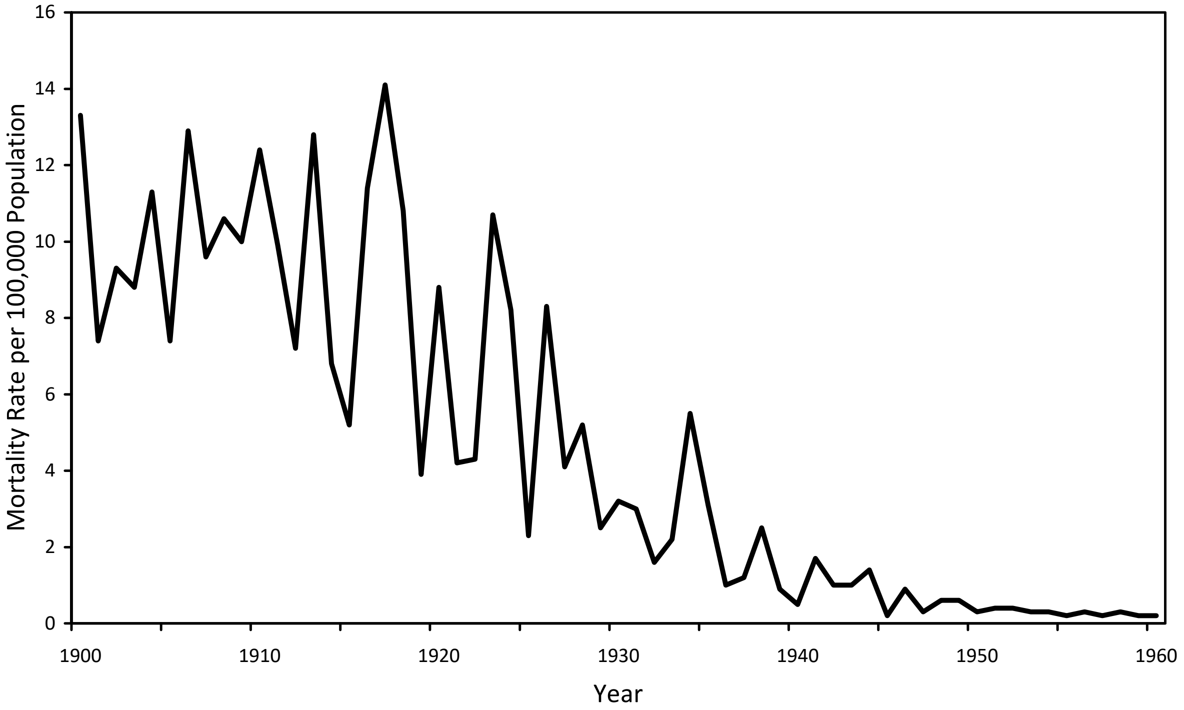

Table S2. Basis for figures concerning mumps

**A. Number of mumps cases, 1963-1966**<br><br>Before licensure of the mumps vaccine, there were an annual 200,000 reported cases of mumps [9]. However, similar to varicella (Table S10), because almost everyone in the population contracted mumps before adulthood [10,11], the number of mumps cases was actually an average of one birth cohort size (4 million cases). Consequently, reported cases comprised only 5% of all cases, and the case fatality rate based on reported cases of mumps (1 in 5,000) [9] is greater than the case fatality rate with respect to all mumps cases.
**B. Estimated number of permanent disabilities from mumps for 2014 in the absence of mass vaccination**<br><br>Although mumps encephalitis can occur in as many as 1 in 300 reported cases, non-fatal permanent injury from mumps encephalitis is very rare [9]. Mumps infection can lead to permanent impaired hearing, and such cases occur one-fourth as often as mumps-related death [9]. Therefore, based on an estimated 43 mumps deaths for 2014, we calculated 11 (≈ 43/4) cases of mumps resulting in permanent impaired hearing. In addition, mumps can permanently impair fertility in post-puberty males. Mumps orchitis occurs in 20% of infected adult males [12] of which 13% suffer impaired fertility [12,13]—a total of 2.6% of mumps-infected adult males. Since impaired hearing occurs in 4.1% of mumps-infected adult males [9], impaired fertility occurs two-thirds (≈ 2.6%/4.1%) as often as impaired hearing in adult males. On this basis, we calculated 7 (≈ 11×[2/3]) cases of mumps resulting in permanent impaired fertility.

Table S3. Basis for figures concerning rubella

**A. Number of rubella cases, 1960-1968**<br><br>Although the CDC did not begin tracking rubella cases until 1966, state governments tracked rubella cases for the entire decade. From 1960 to 1968, there were 1,366,541 reported cases of rubella—152,000 annual reported cases [14,15]. However, similar to varicella (Table S10), because almost everyone in the population contracted rubella before adulthood [14], the number of rubella cases was actually an average of one birth cohort size (4 million cases). Consequently, reported cases comprised only 4% of all cases.
**B. Estimated number of cases of CRS, 1960-1968**<br><br>Because the CDC did not track the number of CRS cases prior to licensure of the rubella vaccine, the number of CRS cases during 1960 to 1968 is unknown; however, it was estimated that there were 20,000 cases of CRS in the 1964-1965 rubella epidemic [14]. In 1969, the CDC began officially tracking CRS cases [15]. Data recorded after vaccine licensure allow for an estimation of the rate of CRS as a function of the number of reported rubella cases; these data indicate that the estimate of 20,000 CRS cases was a significant overestimate [16]. To derive a more accurate estimate, we evaluated the data the CDC gathered in the first few years of CRS tracking. From 1969 to 1971, the CDC recorded 173 cases of CRS and 159,324 reported cases of rubella—1 case of CRS for every 921 reported cases of rubella [15]. From 1960 to 1968, there were 1,366,541 reported cases of rubella (Table S3A). Therefore, during 1960 to 1968, there were an estimated total of 1,484 cases of CRS (= 1,366,541/921).

Table S4. Basis for figures concerning tetanus

**A. Estimated incidence of tetanus, 1943-1945**<br><br>The federal government did not track the number of tetanus cases until 1947 [17]. Because of this, we estimated the number of cases during 1943 to 1945 using the federal health department's tetanus-case statistics recorded between 1947 and 1949. During this period, there were 1,740 reported cases [**Error! Bookmark not defined.**] of which 1,415 died [18–20]—a case fatality rate of 81.3%.<br><br>During 1943 to 1945, annually there were 626 tetanus deaths [21–23], mostly among the population <20 years of age [24]. Based on an 81.3% case fatality rate, we calculated that there were 770 (= 626/81.3%) annual tetanus cases between 1943 and 1945. Since the average population was 138.3 million, there was one case of tetanus for every 180,000 people.
**B. Tetanus case fatality rate among unvaccinated individuals <80 years of age, 2000-2008**<br><br>From 2001 to 2008, more than 75% of the reported tetanus cases whose vaccination status was known were either unvaccinated or had antitoxin levels below the minimal protective level (i.e. had not received a dose of tetanus toxoid in the previous 10 years) [25,26]. In addition, an analysis of these cases showed that vaccination status did not significantly affect the case fatality rate when comparing subjects in the same age groups [25]. Therefore, these cases have the greatest bearing on calculating the tetanus case fatality rate for an unvaccinated population. From 2001 to 2008, there were 233 cases of tetanus resulting in 26 deaths [27]. Individuals <80 years of age accounted for half of those tetanus deaths (13) and 89% of the cases (207) for a case fatality rate of 6.3% (= 13/207) [27].

Figure S2. Decline in tetanus mortality, 1900-1945 [8]

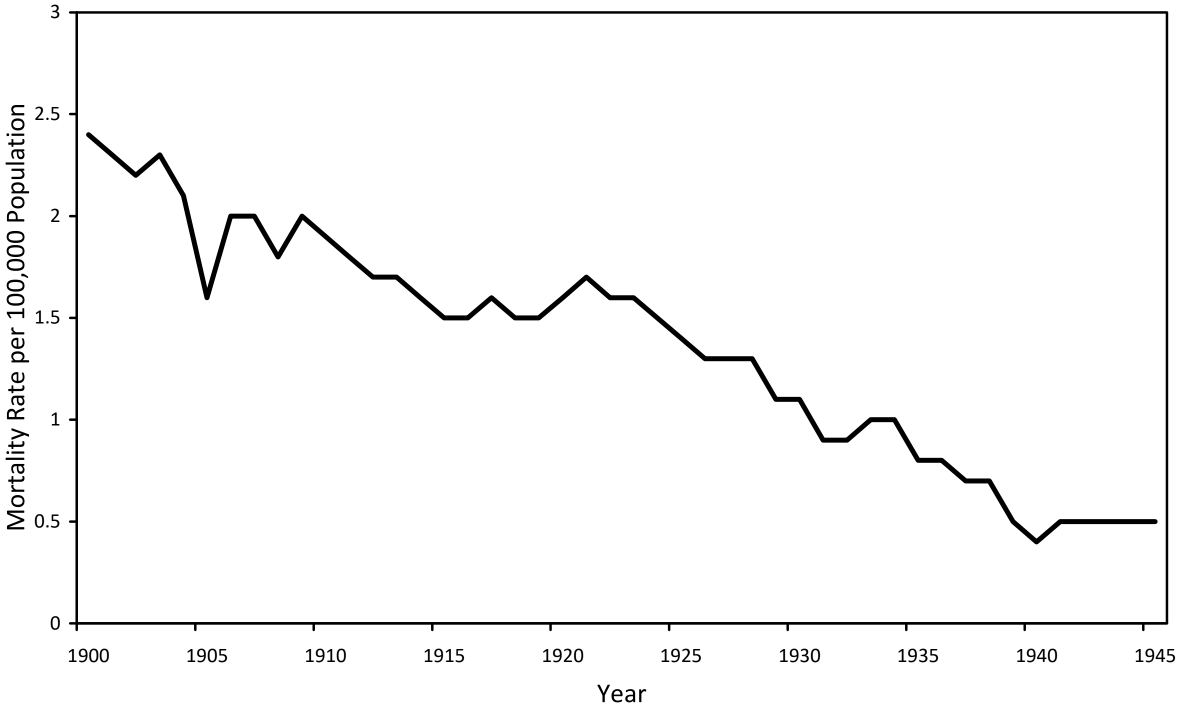

Table S5. Basis for figures concerning diphtheria

**Estimated number of deaths from diphtheria for 2014 in the absence of mass vaccination**
Starting in 1879, there was an exponential decline in the national diphtheria mortality rate. The national decline was stable and did not accelerate until the implementation of the national mass vaccination program in the late 1940s [28,29]. The mortality rate declined from 180.6 per 100,000 population in 1879 to 1.2 per 100,000 population in 1945 [8,30,31]. We approximated the declining rate with the exponential function $R = 41/[1.95^{([Y-1900]/9)}]$, where R is the mortality rate per 100,000 population and Y is the corresponding year. The graphs in Figure S3 show both the recorded rates (bold curve) and the rates approximated by the stated function (thin curve). To calculate the projected mortality rate for 2014 in the absence of mass vaccination, we used the declining trend from 1879 to 1945 to obtain a diphtheria mortality rate of 0.0087 per 100,000. We multiplied the mortality rate of 0.0087 by the population from 2014 (319 million) to obtain 28 deaths, mostly among the population between 1 and 9 years of age [24].

Figure S3. Decline in diphtheria mortality, 1879-1945 [8,30,31]

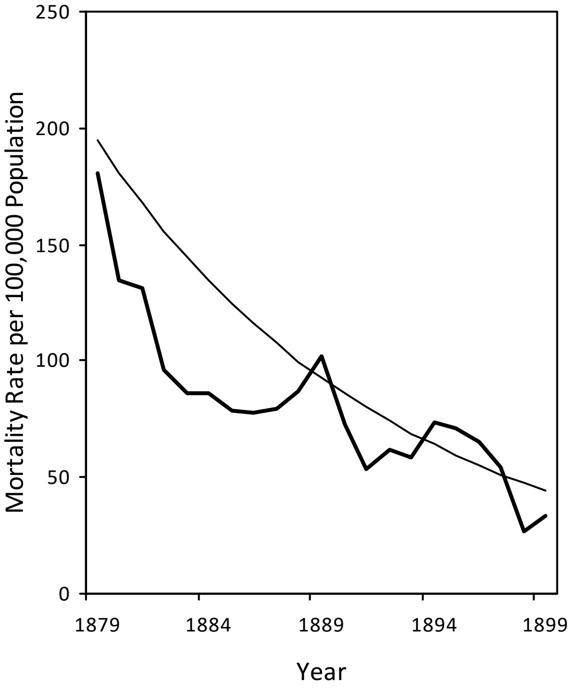

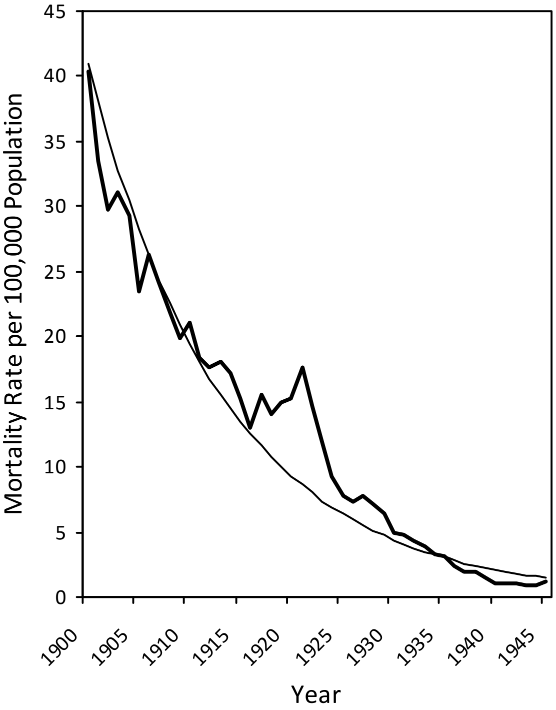

Table S6. Basis for figures concerning pertussis

**A. Estimated number of cases of pertussis for 2014 in the absence of mass vaccination**<br><br>During 1943 to 1945, annually there were 145,185 reported cases of pertussis [17]. Nearly all of those cases occurred in the 25 million children <10 years of age [32], or 1 reported case of pertussis for every 170 children. To estimate the number of reported pertussis cases for 2014 in the absence of mass vaccination, we multiplied the pre-vaccine incidence ratio of 1 in 170 by 2014's population that was <10 years of age (40 million) to obtain 235,000 reported cases. Since pre-vaccine data suggests that only 18% of all cases of pertussis were reported [33], the estimated number of all pertussis cases for 2014 is 1.3 million (≈ 235,000/18%) in the absence of mass vaccination.
**B. Pertussis reported case fatality rate among unvaccinated infants <3 months of age, 2012-2014**<br><br>Most infants <3 months of age were unvaccinated because they could not get the first dose of the vaccine until 2 months of age [34]. The remaining infants, those who were between 2 and 3 months of age, could only receive one shot of the 3-dose series. This single dose was only 50% effective and did not significantly affect the severity of pertussis in those that contracted it [35]. Therefore, pertussis cases in infants <3 months of age have the greatest bearing on estimating the pertussis case fatality rate for a hypothetically unvaccinated population in 2014. From 2012 to 2014, annually there were 1,750 reported cases of pertussis among infants <3 months of age resulting in 12 deaths—a reported case fatality rate of 0.7% [36–38].

C. Estimated number of cases of pertussis among infants <3 months of age for 2014 in the absence of mass vaccination
To estimate the number of pertussis cases <3 months of age for 2014 in the absence of mass vaccination, we used the incidence of pertussis in that cohort prior to mass vaccination. In the 1930s, 7.5% of all reported cases of pertussis occurred in infants <1 year of age [32]. Since those cases were evenly distributed throughout the year [32], 1.9% (≈ 7.5%/4) of all reported cases of pertussis occurred in infants <3 months of age. Therefore, 4,500 (1.9%) of the estimated 235,000 reported cases of pertussis for 2014 would occur in infants <3 months of age.
**D. Number of pertussis deaths and percentage that occurred among infants <3 months of age, 1943-1945**
During 1943 to 1945, annually there were 2,300 pertussis deaths of which 600 (≈ 26% of 2,300) occurred in infants <3 months of age [21–23,39–41].

Figure S4. Decline in pertussis mortality, 1900-1945 [Error! Bookmark not defined.**]**

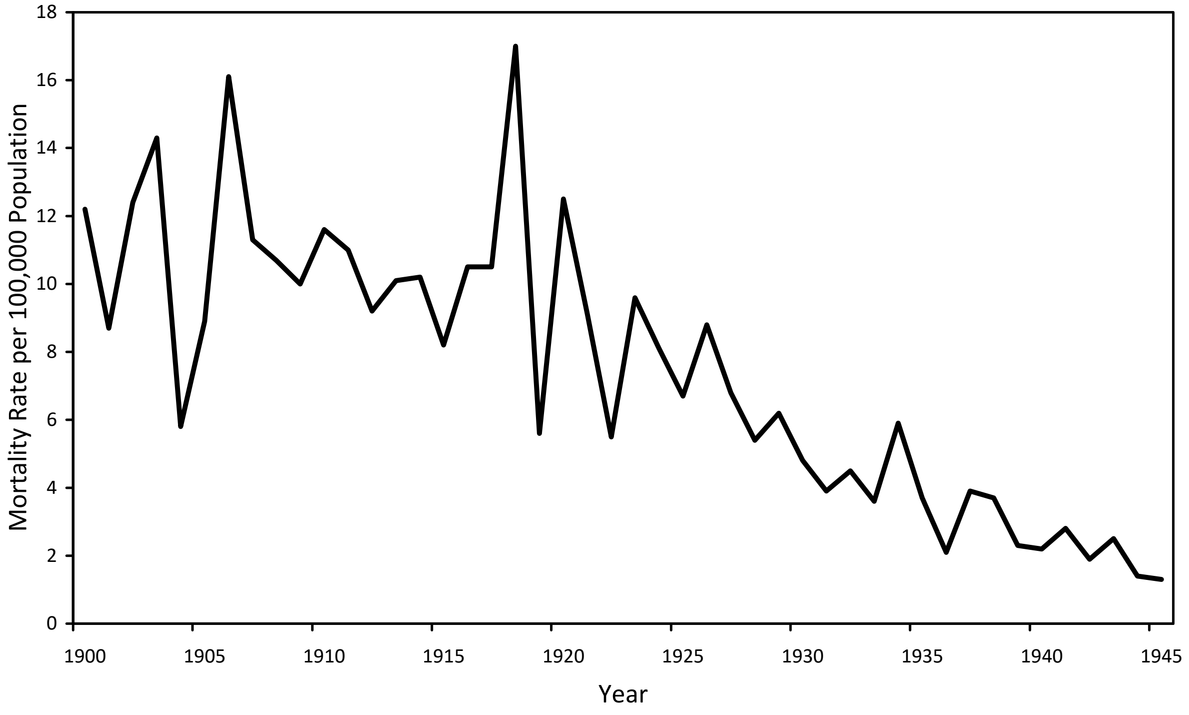

Table S7. Basis for figures concerning polio

A. Estimated number of cases of paralytic poliomyelitis, 1935-1954

During 1935 to 1954, the average reported polio mortality rate was 0.95 per 100,000 population [8]. However, in this time period, the broad criteria used to diagnose paralytic poliomyelitis resulted in over-reporting of cases. After 1955, diagnostic criteria were revised to better exclude false positives; laboratory confirmation and the presence of long-term residual paralysis were introduced as new conditions for diagnosis. In 1959, a re-analysis of the reported cases of paralytic poliomyelitis from 1951 to 1954 using the revised diagnostic criteria concluded that 60% of those cases were false positives and recorded an annual average of 9,000 paralytic poliomyelitis cases [42]. Because the re-analysis studied the four years of a polio epidemic, when the polio mortality rate was 1.24 times greater than the rate recorded during the reference years (1.18 compared to 0.95) [8], we scaled the number of cases recorded in the re-analysis down by 1.24 to obtain an annual average of 7,260 (≈ 9,000/1.24) paralytic poliomyelitis cases.

B. Estimated number of poliomyelitis cases resulting in permanent disability or death, 1935-1954

We used a 1953 study of 1,523 poliomyelitis cases in New York to estimate the percentage of cases that resulted in permanent disability [43]. 616 of the cases in the study had no paralysis, 252 were paralytic with bulbar involvement, and 655 were paralytic with no bulbar involvement. 542 cases had long-term residual paralysis (i.e. were still paralytic after 3 months from onset of illness). After 2 years from onset of illness, 42 bulbar paralytic cases (16.5% of 252) and 223 non-bulbar paralytic cases (34% of 655) were still paralytic—a total of 265 cases. Thus, bulbar cases comprised 15.8% (42/265) of all cases that were paralytic after 2 years from onset of illness. In addition, 1/6 of the cases that were paralytic after 2 years from onset of illness were severely paralyzed (i.e. were disabled by the paralysis). Therefore, we estimated that permanent disability occurred in 8.1% (265/[6*542]) of poliomyelitis cases with long-term residual paralysis.

Post-polio syndrome can also result in permanent disability. A 1992 study of 551 poliomyelitis survivors in Pittsburgh found that post-polio syndrome increased the number of patients with severe impairment from 150 to 166, an increase of 10.7% [44]. Consequently, we raised the estimated percentage of poliomyelitis cases resulting in permanent disability by 10.7% to calculate a rate of 9% (8.1%*110.7%).

Of the estimated 7,260 annual cases of paralytic poliomyelitis during 1935 to 1954, an estimated 530 cases were fatal (Table S7C) and 606 (9% of [7,260–530]) resulted in permanent disability for a total of 1,136 (530+606) cases of death or permanent disability. Because 78% of paralytic poliomyelitis deaths [45] and 15.8% of the permanent disabilities had bulbar involvement, we estimated 509 ([78% of 530]+[15.8% of 606]) deaths and permanent disabilities with bulbar involvement and 627 (1,136–509) such cases without bulbar involvement.

C. Estimated case fatality rate of paralytic poliomyelitis, 1935-1954

To estimate the percentage of paralytic cases that resulted in death during 1935 to 1954, we used the average value recorded between 1956 and 1959, 7.3% [15]. More than 75% of the paralytic cases in that time period were unvaccinated, and most of the remaining 25% were under-vaccinated and suffered paralysis as severely as the unvaccinated group [42,46]. Therefore, we estimated that the case fatality rate of 7.3% was most likely the same in the early 1950s and that there were 530 (≈ 7.3% of 7,260) annual fatal cases of paralytic poliomyelitis.

D. Estimated number of poliomyelitis cases resulting in death or permanent disability for 2014 in the absence of mass vaccination

During 1935 to 1954, almost all poliomyelitis deaths occurred in the population <40 years of age [47], therefore our calculations are focused on this age group. We estimated that permanent disability or death occurred in 1 in 190,000 (134 in 25.4 million) children <10 years of age with tonsils who rested after the onset of significant symptoms and in 1 in 25,000 (302 in 7.6 million) of the remaining children <10 years of age (Table S7E). We also estimated that permanent disability or death occurred in 1 in 383,000 (89 in 34.1 million) individuals 10-39 years of age with tonsils who rested after the onset of significant symptoms and in 1 in 59,000 (611 in 36 million) of the remaining individuals 10-39 years of age (Table S7F).

In 2014, 8% of the adult population had no tonsils [48]—38.5% (8%/20.8%) of the proportion of adults that had no tonsils during 1935 to 1954 (Table S7G). Therefore, we estimated that 4.6% (38.5% of 12%) of the child population had no tonsils in 2014 (Table S7G). Assuming that the same proportion of children <10 years of age in 2014 would rest after significant onset of symptoms as children during 1935 to 1954 (87.5%, Table S7H), we calculated 33.4 million children ([100%-4.6%]*87.5%*40 million) <10 years of age in 2014 with tonsils and rest and 6.6 million children (40 million – 33.4 million) at elevated risk. To estimate the number of poliomyelitis cases resulting in death or permanent disability in the population <10 years of age for 2014 in the absence of mass vaccination, we multiplied the preceding 2014 population sizes by their corresponding pre-vaccine ratios. Thus, children <10 years of age with tonsils and rest would contribute 176 cases of permanent disability or death (≈ 33.4 million/190,000) compared to 264 such cases in children <10 years of age at elevated risk (≈ 6.6 million/25,000).

Assuming that the same proportion of individuals 10-39 years of age in 2014 would rest after significant onset of symptoms as individuals during 1935 to 1954 (61.4%, Table S7H), we calculated 67.8 million individuals (([100%-8%]*61.4%*120 million) 10-39 years of age in 2014 with tonsils and rest and 52.2 million individuals (120 million – 67.8 million) at elevated risk. To estimate the number of poliomyelitis cases resulting in death or permanent disability in the population 10-39 years of age for 2014 in the absence of mass vaccination, we multiplied the preceding 2014 population sizes by their corresponding pre-vaccine ratios. Thus, individuals 10-39 years of age with tonsils and rest would contribute 177 cases of permanent disability or death (≈ 67.8 million/383,000) compared to 885 such cases in individuals 10-39 years of age at elevated risk (≈ 52.2 million/59,000).

Combining the totals for children <10 years of age with those for individuals 10-39 years of age results in 353 (= 176+177) cases of death or permanent disability in the population with tonsils that would rest after significant onset of symptoms and 1,149 (= 264+885) cases in the population at elevated risk.

E. Estimated risk of permanent disability or death from poliomyelitis in children <10 years of age based on tonsillectomy and rest status, 1935-1954

Children <10 years of age comprised 56.4% of all paralytic poliomyelitis cases [49]and 5% of those cases were fatal [50]. Therefore, fatalities among children <10 years of age comprised 2.8% (56.4%*5%) of all paralytic poliomyelitis cases and 38.4% (2.8%/7.3%) of all paralytic poliomyelitis deaths (Table S7C). Since the age distribution of cases of paralytic poliomyelitis resulting in severe impairment was similar to the age distribution of paralytic poliomyelitis fatalities [51], we estimated that children <10 years of age comprised 38.4% of all paralytic poliomyelitis deaths and permanent disabilities.

Of the estimated 509 annual paralytic poliomyelitis cases with bulbar involvement resulting in death or permanent disability during 1934 to 1954 (Table S7B), we calculated 195 (38.4% of 509) occurring in children <10 years of age of which 35.7% (100%–64.3%) had tonsils (Table S7G) and 87.5% rested after

significant onset of symptoms (Table S7H). Therefore, we calculated 61 (195*35.7%*87.5%) cases with bulbar involvement among children <10 years of age that had tonsils and rested. Of the estimated 627 annual paralytic poliomyelitis cases without bulbar involvement resulting in death or permanent disability (Table S7B), we calculated 241 (38.4%) occurring in children <10 years of age of which 83.5% (100%–16.5%) had tonsils [49] and 36.4% rested (Table S7H). Therefore, we calculated 73 (241*83.5%*36.4%) cases without bulbar involvement among children <10 years of age that had tonsils and rested. The combined number of estimated deaths and permanent disabilities from paralytic poliomyelitis among children <10 years of age is 134 (61+73) out of 25.4 million (33 million*[100%–12%]*87.5%) children with tonsils that rested (Table S7G-H) and 302 (195+241–134) out of 7.6 million (33 million–25.4 million) children at elevated risk.

F. Estimated risk of permanent disability or death from poliomyelitis in individuals 10-39 years of age based on tonsillectomy and rest status, 1935-1954

Of the estimated 509 annual paralytic poliomyelitis cases with bulbar involvement resulting in death or permanent disability during 1934 to 1954 (Table S7B), we calculated 314 (509–195) occurring in individuals 10-39 years of age (Table S7E) of which 22.4% (100%–77.6%) had tonsils (Table S7G) and 61.4% rested after significant onset of symptoms (Table S7H). Therefore, we calculated 43 (314*22.4%*61.4%) cases with bulbar involvement among individuals 10-39 years of age that had tonsils and rested. Of the estimated 627 annual paralytic poliomyelitis cases without bulbar involvement resulting in death or permanent disability (Table S7B), we calculated 386 (627–241) occurring in individuals 10-39 years of age (Table S7E) of which 54.2% (100%–45.8%) had tonsils [49] and 21.8% rested (Table S7H). Therefore, we calculated 46 (386*54.2%*21.8%) cases without bulbar involvement among individuals 10-39 years of age that had tonsils and rested. The combined number of estimated deaths and permanent disabilities from paralytic poliomyelitis among individuals 10-39 years of age is 89 (43+46) out of 34.1 million (70.1 million*[100%–20.8%]*61.4%) individuals with tonsils that rested (Table S7G-H) and 611 (314+386–89) out of 36 million (70.1 million–34.1 million) individuals at elevated risk.

G. Percentage of population that had no tonsils, 1935-1954

Among individuals born between 1930 and 1936, 31.9% had no tonsils [52]. A 1946 study showed that 86.1% of paralytic poliomyelitis cases with bulbar involvement in that age group had no tonsils [49], therefore we computed an odds ratio [53] of 13.2 ([(100%-31.9%)×86.1%]/[31.9%×(100%-86.1%)]) for that age group. Among paralytic poliomyelitis cases with bulbar involvement <10 years of age, 64.3% had no tonsils [49]. Presuming that the odds ratio was also 13.2 among children <10 years of age, we solved the equation ([1–P]×64.3%)/(P×[100%-64.3%]) = 13.2 where P is the percentage of all children <10 years of age with no tonsils to obtain 12%. Among paralytic poliomyelitis cases with bulbar involvement ≥10 years of age, 77.6% had no tonsils [49]. Presuming that the odds ratio was also 13.2 among all individuals ≥10 years of age, we solved the equation ([1–P]×77.6%)/(P×[100%-77.6%]) = 13.2 where P is the percentage of all individuals ≥10 years of age with no tonsils to obtain 20.8%.

H. Percentage of poliomyelitis cases that rested after significant onset of symptoms by type of paralysis and severity, 1935-1954

We used a 1950 study of 411 poliomyelitis cases in Los Angeles, North Carolina, and New York to estimate the percentage of poliomyelitis cases that rested after significant onset of symptoms by type of paralysis and severity [54]. Among poliomyelitis cases without bulbar involvement in the <10 age group, 87.5% of mild cases and 36.4% of severe cases rested. Among poliomyelitis cases without bulbar involvement in the 10-14 age group, 79.4% of mild cases and 25% of severe cases rested. Among poliomyelitis cases without bulbar involvement in the >14 age group, 47.8% of mild cases and 20% of

severe cases rested [54]. Because the 10-14 age group comprised 43% of mild cases and 36.2% of severe cases without bulbar involvement in the ≥10 age group [49], we calculated that 61.4% (43%*79.4% + [100%-43%]*47.8%) of mild cases and 21.8% (36.2%*25% + [100%-36.2%]*20%) of severe cases rested in the ≥10 age group.

Resting after significant onset of symptoms had no effect on the severity of poliomyelitis cases with bulbar involvement [54]. Consequently, we estimated that the percentage of cases with bulbar involvement that rested was the same as the percentage of all poliomyelitis cases that rested. Since more than 99% of poliomyelitis cases did not result in severe paralysis [50], we estimated that the rates of rest observed in the population with mild cases were representative of the population as a whole. Therefore, we estimated that 87.5% of poliomyelitis cases with bulbar involvement in the <10 age group and 61.4% of such cases in the ≥10 age group rested after significant onset of symptoms.

Table S8. Basis for figures concerning *Haemophilus influenzae* type b

A. Estimated number of cases of invasive Hib in the absence of mass vaccination, 1994-2000

Because the CDC did not track the number of invasive Hib cases during the reference years [15], the number of invasive Hib cases in this time period is unknown. However, it was estimated that annually there were 20,000 cases of invasive *H. influenzae* in children <5 years of age, 19,000 type b and 1,000 non-type b (presuming that non-type b cases comprised 5% of all invasive *H. influenzae* in children <5 years of age) [55,56]. In the 1990s, the CDC began tracking invasive *H. influenzae* cases of all types [15]. Data recorded in the 1990s showed that the previous estimate of 20,000 invasive cases was a significant overestimate. To derive a more accurate estimate, we considered the data the CDC gathered from 1994 to 2000. In this time period, annually there were 180 cases of non-type b invasive *H. influenzae* in children <5 years of age (Table S8B). On the basis that the first few years of mass vaccination did not affect the incidence of non-type b invasive *H. influenzae* and that non-type b cases comprised 5% of all cases before mass vaccination, we estimated that 3,600 (= 180/5%) annual cases of all types would have occurred during 1994 to 2000 in the absence of mass vaccination, and type b would have comprised 3,400 (≈ 95%) of those cases.

B. Invasive *H. influenzae* tracking in children <5 years of age, 1994-2000 [57,58]

From 1994 to 1995, there were 669 reported cases of invasive *H. influenzae*. The serotype was known for 376 (56%) of those cases and 192 (51% of known cases) were non-type b. Therefore, there were an estimated 341 (= 51% of 669) non-type b cases. From 1996 to 2000, there were 1,360 reported cases of invasive *H. influenzae* (272 per year). The serotype was known for 1,034 (76%) of those cases and 693 (67% of known cases) were non-type b. Therefore, there were an estimated 911 (= 67% of 1,360) non-type b cases. The total estimated number of non-type b cases from 1994 to 2000 was 1,252 (= 341+911) or 180 (≈ 1,252/7) cases per year.

C. Estimated number of cases of invasive Hib resulting in death or permanent disability in the absence of mass vaccination, 1994-2000

60% of invasive Hib cases result in meningitis, frequently accompanied by bacteraemia. 11% of those cases result in permanent neurologic sequelae such as deafness and intellectual impairment, and an additional 5% of those cases are fatal [59]. 15% of invasive Hib cases result in epiglottitis, and 1% of those cases are fatal [59,60]. Therefore, the estimated annual total of Hib-related combined deaths and permanent disabilities during 1994 to 2000 in the absence of mass vaccination is 330 (≈ 3,400×[(60%×[11%+5%])+(15%×1%)]).

D. Protective effect of breastfeeding against invasive Hib measured in 1997 Swedish study of children <6 years of age [61]

Of the children who had contracted Hib at any point in their life, 37% had been breastfed exclusively for ≥13 weeks, 63% were not. Of the children in the control group (who had never contracted Hib), 62% had been breastfed exclusively ≥13 weeks, 38% were not. The statistical odds ratio [53] is 2.8 (= [62%×63%]/[38%×37%]).

E. Estimated number of cases of invasive Hib resulting in death or permanent disability for 2014 in the absence of mass vaccination based on breastfeeding status in the United States

During 1994 to 2000, had mass vaccination not been implemented, we estimated the incidence of permanent injury from invasive Hib to be 1 in 143,000 for children that were breastfed exclusively for ≥13 weeks in the United States and 1 in 51,000 for children that were breastfed exclusively for <13 weeks (Table S8F). To estimate the number of invasive Hib cases for 2014 resulting in death or permanent disability in the absence of mass vaccination, we multiplied those 1990s ratios by the 2014 population <5 years of age that was breastfed exclusively for ≥13 weeks in the United States (47% of 20 million) and the 2014 population <5 years of age that was breastfed exclusively for <13 weeks (53% of 20 million) [62]. Thus, children that were breastfed exclusively for ≥13 weeks would contribute 66 cases of permanent injury (= 47% of 20 million/143,000) compared to 208 cases of permanent injury in children that were breasted for <13 weeks (= 53% of 20 million/51,000).

F. Estimated risk of invasive Hib based on breastfeeding status in the United States in the absence of mass vaccination, 1994-2000 [62,63]

In the late 1990s, 60% of children in the United States were breastfed and 40% of those children were exclusively breastfed through 3 months—24% (= 60%×40%) of all children. On the basis that children that were breastfed exclusively for <13 weeks outnumbered children that were breastfed exclusively for ≥13 weeks by a factor of 3 to 1 and were 2.8 times more likely to contract invasive Hib, we calculated that, of the estimated 330 annual cases of invasive Hib resulting in permanent injury, 35 cases would probably have occurred in children that were breastfed for ≥13 weeks compared to 295 (≈ 35×2.8×3) cases in children that were breastfed for <13 weeks. Since the average population <5 years of age from 1994 to 2000 was 20 million, we estimated the incidence of permanent injury from invasive Hib to be 1 in 143,000 (≈ [1/4]×[20 million/35]) for children that were breastfed for ≥13 weeks and 1 in 51,000 (≈ [3/4]×[20 million/295]) for children that were breastfed for <13 weeks.

Table S9. Basis for figures concerning hepatitis B

A. Estimated number of cases of hepatitis B for 2014 in the absence of mass vaccination

During the reference years 1988 to 1990, annually there were an average of 22,566 reported hepatitis B cases [15] out of an average population of 250 million (≈ 1 in 11,100). To estimate the number of reported cases of hepatitis B for 2014 in the absence of mass vaccination, we multiplied the pre-vaccine incidence ratio of 1/11,100 by 2014's population (319 million) to obtain 29,000 reported cases. Since the CDC estimates that only 1 in 6.5 hepatitis B cases were reported [64], for 2014 in the absence of mass vaccination, the estimated number of all cases of hepatitis B is 190,000 (≈ 29,000×6.5).

B. Estimated number of cases of fatal fulminant hepatitis B for 2014 in the absence of mass vaccination

Annually, because 1% of reported cases (not total cases) of hepatitis B lead to fatal fulminant hepatitis, the estimated number of fatal fulminant hepatitis cases for 2014 is 300 (≈ 1% of 29,000)—nearly all occurring in adults and adolescents [65].

C. Estimated number of hepatitis B cases leading to death from chronic infection before age 80 for 2014 in the absence of mass vaccination among various age groups

92% of hepatitis B infections occur in adults and adolescents [66], 5% of those infections develop into chronic infections, and 15% of those chronic infections lead to death later in life [65]. We multiplied the product of those percentages (92%×5%×15%) by the estimated 190,000 cases of hepatitis B for 2014 to obtain 1,311 fatal cases. We estimated that 1,100 (≈ 85% of 1,311) [67] of those fatal cases would occur in individuals <80 years of age.

4% of hepatitis B infections occur after birth in children ≤10 years of age [66], 18% of those infections develop into chronic infections (30% for children <5 years of age and 5% for the remaining children results in an average of 18%), and 25% of those chronic infections lead to death later in life [65]. We multiplied the product of those percentages (4%×18%×25%) by the estimated 190,000 cases of hepatitis B for 2014 to obtain 342 fatal cases. We estimated that 290 (≈ 85% of 342) [67] of those fatal cases would occur in individuals <80 years of age.

4% of infections occur in children at birth [66], 90% of those infections develop into chronic infections, and 25% of those chronic infections lead to death later in life [65]. We multiplied the product of those percentages (4%×90%×25%) by the estimated 190,000 cases of hepatitis B for 2014 to obtain 1,710 fatal cases. We estimated that 1,450 (≈ 85% of 1,710) [67] of those fatal cases would occur in individuals <80 years of age.

Combining the estimated fatal cases for children infected at birth with those for children infected after birth results in 1,740 (= 290+1,450) fatal cases from childhood hepatitis B infection.

D. Estimated number of hepatitis B cases leading to death before age 80 for 2014 in the absence of mass vaccination among adults and adolescents at high risk of exposure

Among adults and adolescents, 79% of cases occur in individuals who have sex with an infected partner, heterosexuals with multiple sex partners, men who have sex with men, and injection-drug users [66]. Most of the remaining 21% of cases are due to other unidentified sources. A 1990 study found that those

patients with no identifiable source of their infection dwelled in communities where hepatitis B was three times more prevalent than it was in the general population [68]. Consequently, we estimated that 7% (= 21%/3) of cases occur in communities at normal risk. Of the estimated 1,400 deaths among adults and adolescents (300 deaths from fulminant hepatitis + 1,100 deaths from chronic infection) for 2014 in the absence of mass vaccination, we obtained 100 (≈ 7% of 1,400) deaths among individuals at normal risk and 1,300 deaths among individuals at high risk.

E. Estimated number of hepatitis B cases leading to death before age 80 for 2014 in the absence of mass vaccination among children at high risk of exposure

Among children, all infections occurring at birth originate from mothers who were already chronically infected. Studies of highly endemic Asian communities in the US in the early 1990s showed that two-thirds of children infected after their birth dwelled with one or more chronically infected individuals [69]. Because the remaining third of those children dwelled in communities where hepatitis B was 15 times more prevalent than it was in the general population [70], we estimated that 2.2% (= 33%/15) of cases occur in communities at normal risk. Of the estimated 290 deaths among children infected after birth for 2014 in the absence of mass vaccination, we obtained 6 (≈ 2.2% of 290) deaths among children at normal risk. We added the remaining 284 (= 290–6) deaths from infections after birth to the estimated 1,450 deaths from infections at birth to obtain 1,734 deaths among children at high risk.

Table S10. Basis for figures concerning varicella

Number of varicella cases, 1991-1994

Before licensure of the varicella vaccine, there were an annual 150,000 reported cases of varicella [15]. However, the CDC states, "In the prevaccine era, varicella was endemic in the United States, and virtually all persons acquired varicella by adulthood. As a result, the number of cases occurring annually was estimated to approximate the birth cohort, or approximately 4 million per year [71]." Consequently, reported cases comprised only 4% of all cases.

Table S11. Basis for figures concerning human papillomavirus

A. Estimated number of HPV cases leading to death before age 80 for 2014 among women
Among women, HPV can lead to cancers of the cervix, vagina, vulva, anus, rectum, and oropharynx. Cervical cancer is the most common and comprises 53% of HPV-attributable cancers targeted by vaccines in women [72]. Annually there are 9,400 cases of cervical cancer targeted by vaccines (≈ 80% of 11,700 total cases) [72] resulting in 2,900 deaths (≈ 80% of 3,650 total deaths) in women <80 years of age [73].<br><br>Women that are not screened with a Pap or HPV test every three years are significantly more likely to die from cervical cancer. Pap tests are able to detect 88% of cervical cancers, and if used together with HPV testing, the success rate is 95% [74]. Since half of women that are tested use both Pap and HPV testing, 92% (= [88%+95%]/2) of all cervical cancers in women that are tested are detected, and those women can have the cancerous tissue safely removed before it spreads. Most cases of cervical cancer occur in women that are underscreened. 50% of women with cervical cancer have never been tested, and 20% go more than three years without testing [75]. Only the remaining 30% of cervical cancer cases occur in women that are tested every three years, and 92% of those cases are detected early by testing and are addressed.<br><br>Using the preceding percentages, we calculated that, of the 9,400 annual cases of cervical cancer targeted by vaccines in women <80 years of age between 2011 and 2014, 6,580 (= 70% of 9,400) are underscreened, 2,595 (= 30%×92%×9,400) are successfully screened, and 225 (= 30%×[100%–92%]×9,400) are screened with false negatives. Since 3.3% (= 225 /[6,580+225]) of undetected cervical cancer cases are in women that are tested every three years, 96 of the fatal cases (= 3.3% of 2,900) are most likely in this group.<br><br>In addition, women that smoke are two times more likely to develop cervical cancer [76]. Since 15.3% of women smoke [77], non-smoking women outnumber smoking women by a factor of 5.5 to 1 (≈ 84.7%/15.3%). For every 5.5 non-smoking women that develop cervical cancer, there are two smoking women that develop cervical cancer—27% of cervical cancer cases (= 2/[5.5+2]). Consequently, we calculated that, of the estimated 96 annual fatal cases of cervical cancer targeted by vaccines and occurring in women <80 years of age that are tested every three years between 2011 and 2014, 70 (= 73% of 96) cases occur in women that do not smoke. In comparison, an estimated 2,830 (= 2,900–70) fatal cases of cervical cancer occur in women that either smoke or are not screened every three years. Since cervical cancer comprises 53% of all HPV-attributable cancers targeted by vaccines in women, we estimated 132 (= 70/53%) fatal HPV-attributable cancers occurring in women at normal risk and 5,340 (= 2,830/53%) fatal cancers occurring in women at high risk.
**B. Estimated number of HPV cases leading to death before age 80 for 2014 among men**
Among men, HPV can lead to cancers of the oropharynx, penis, anus, and rectum. Oropharyngeal cancer is the most common and comprises 80% of HPV-attributable cancers targeted by vaccines in men [72]. Annually there are 9,000 cases of oropharyngeal cancer targeted by vaccines (≈ 68% of 13,300 total cases) [72]. 450 (5%) of those cases are fatal [78], and 350 (78%) of those fatal cases occur in men <80 years of age [79].

Men that have smoked or have had ≥6 oral sex partners in their lifetime are significantly more likely to die from oropharyngeal cancer. Of the 350 fatal oropharyngeal cases, 98 cases (28%) occur in men that have never smoked [80], and 53 (54%) of those cases occur in men that have had <6 oral sex partners in their lifetime [81]. Since oropharyngeal cancer comprises 80% of all HPV-attributable cancers targeted by vaccines in men, we estimated 66 (= 53/80%) fatal HPV-attributable cancers occurring in men at normal risk and 371 (= [350-53]/80%) fatal cancers occurring in men at high risk.

References

1. Centers for Disease Control and Prevention. Measles prevention: Recommendations of the Immunization Practices Advisory Committee (ACIP). *Morb. Mortal. Wkly. Rep.* **1989**, *38*, 1–18.
2. Centers for Disease Control and Prevention. Complications of Measles. Available online: https://www.cdc.gov/measles/symptoms/complications.html (accessed on 29 January 2020).
3. Centers for Disease Control and Prevention. Measles. In *Epidemiology and Prevention of Vaccine-Preventable Diseases*, 13th ed.; Hamborsky, J., Kroger, A., Wolfe, S., Eds.; Public Health Foundation: Washington, DC, USA, 2015; pp. 209–230.
4. World Health Organization: M-M-R II (Measles, Mumps, and Rubella Virus Vaccine Live). Whitehouse Station: Merck & Co., Inc.; 2015 Available online: https://www.who.int/immunization_standards/vaccine_quality/PQ_168_MMR_MSD_PI_July2008.pdf (accessed on 22 October 2016).
5. Hussey, G.D.; Klein, M. A randomized, controlled trial of vitamin A in children with severe measles. *N. Engl. J. Med.* **1990**, *323*, 160–164.
6. Frieden, T.R.; Sowell, A.L.; Henning, K.J.; Huff, F.L.; Gunn, R.A. Vitamin A Levels and Severity of Measles: New York City. *Am. J. Dis. Child.* **1992**, *146*, 182–186.
7. Butler, J.C.; Havens, P.L.; Sowell, A.L.; Huff, D.L.; Peterson, D.E.; Day, S.E.; Bennin, R.A.; Circo, R.; Davis, J.P. Measles severity and serum retinol (vitamin A) concentration among children in the United States. *Pediatrics* **1993**, *91*, 1176–1181.
8. United States Department of Health, Education, and Welfare. *Death Rates for Detailed Causes: Death-Registration States, 1900–1932, and United States, 1933–1960*; Vital Statistics Rates in the United States 1940–1960; U.S. Government Printing Office: Washington, DC, USA, 1968; pp. 559–603.
9. Centers for Disease Control and Prevention. Mumps. In *Epidemiology and Prevention of Vaccine-Preventable Diseases*, 13th ed.; Hamborsky, J., Kroger, A., Wolfe, S., Eds.; Public Health Foundation: Washington, DC, USA, 2015; pp. 247–260.
10. World Health Organization. Mumps Virus Vaccines. *Wkly. Epidemiol. Rec.* **2007**, *82*, 51–60.
11. Wagenvoort, J.H.; Harmsen, M.; Boutahar-Trouw, B.J.; Kraaijeveld, C.A.; Winkler, K.C. Epidemiology of mumps in the Netherlands. *J. Hyg.* **1980**, *85*, 313–326.
12. Werner, C.A. Mumps Orchitis and Testicular Atrophy. II. A Factor in Male Sterility. *Ann. Intern. Med.* **1950**, *32*, 1075–1086.
13. Davis, N.F.; McGuire, B.B.; Mahon, J.A.; Smyth, A.E.; O'Malley, K.J.; Fitzpatrick, J.M. The increasing incidence of mumps orchitis: A comprehensive review. *Br. J. Urol. Int.* **2010**, *105*, 1062–1065.
14. National Communicable Disease Center. *Rubella Surveillance, June 1969*; National Communicable Disease Center: Atlanta, GA, USA, 1969; pp. 1-14.
15. Centers for Disease Control and Prevention. Reported Cases and Deaths from Vaccine Preventable Diseases, United States, 1950–2013. In *Epidemiology and Prevention of Vaccine-Preventable Diseases*, 13th ed.; Hamborsky, J., Kroger, A., Wolfe, S., Eds.; Public Health Foundation: Washington, DC, USA, 2015; pp. E1–E6.
16. Lockett, T.; Rudolph, J. Deaf-blind children with maternal rubella: Implications for adult services. *Am. Ann. Deaf* **1980**, *12*, 1002–1006.
17. Centers for Disease Control and Prevention. Annual Summary 1980: Reported Morbidity & Mortality in the United States. *Morb. Mortal. Wkly. Rep.* **1981**, *29 Pt 1*, 1–17.

18. United States Public Health Service. Table 22—Deaths from each cause, by race and sex: United States and each State. In *Vital Statistics of the United States 1947 Part 2*; United States Government Printing Office: Washington, DC, USA, 1949; pp. 444–511.
19. United States Public Health Service. Table 22—Deaths from each cause, by race and sex: United States and each State. In *Vital Statistics of the United States 1948 Part 2*; United States Government Printing Office: Washington, DC, USA, 1950; pp. 440–507.
20. United States Public Health Service. Table 23—Deaths from 254 selected causes, by race and sex: United States and each State. In *Vital Statistics of the United States 1949 Part 2*; United States Government Printing Office: Washington, DC, USA, 1951; pp. 458–525.
21. United States Bureau of the Census. Table 24—Deaths from each cause, by race and sex: United States and each State. In *Vital Statistics of the United States 1943 Part 2*; United States Government Printing Office: Washington, DC, USA, 1945; pp. 498–561.
22. United States Bureau of the Census. Table 24—Deaths from each cause, by race and sex: United States and each State. In *Vital Statistics of the United States 1944 Part 2*; United States Government Printing Office: Washington, DC, USA, 1946; pp. 482–549.
23. United States Public Health Service. Table 20—Deaths from each cause, by race and sex: United States and each State. In *Vital Statistics of the United States 1945 Part 2*; United States Government Printing Office: Washington, DC, USA, 1947; pp. 378–445.
24. United States Public Health Service. Table 5—Deaths from each cause, by age, race, and sex: United States, 1945. In *Vital Statistics of the United States 1945 Part 1*; United States Government Printing Office: Washington, DC, USA, 1947; pp. 54–101.
25. Centers for Disease Control and Prevention. Tetanus Surveillance—United States, 2001–2008. *Morb. Mortal. Wkly. Rep.* **2011**, *60*, 365–369.
26. Centers for Disease Control and Prevention. Tetanus. In *Epidemiology and Prevention of Vaccine-Preventable Diseases*, 13th ed.; Hamborsky, J., Kroger, A., Wolfe, S., Eds.; Public Health Foundation: Washington, DC, USA, 2015; pp. 341–352.
27. Centers for Disease Control and Prevention. *Chapter 16: Tetanus. Manual for the Surveillance of Vaccine-Preventable Diseases*, 5th ed.; Centers for Disease Control and Prevention: Atlanta, GA, USA, 2011; pp. 1–6.
28. Lee, W.W. The Factor of Chance in Diphtheria Mortality. *Am. J. Public Health Nation's Health* **1929**, *19*, 855–862.
29. Centers for Disease Control and Prevention. Diphtheria. In *Epidemiology and Prevention of Vaccine-Preventable Diseases*, 13th ed.; Hamborsky, J., Kroger, A., Wolfe, S., Eds.; Public Health Foundation: Washington, DC, USA, 2015; pp. 107–118.
30. United States Department of Health, Education, and Welfare. Figure 18—Death Rates for Diphtheria: Death-registration States, 1900–1932, and United States, 1933–1960. In *Vital Statistics Rates in the United States 1940–1960*; U.S. Government Printing Office: Washington, DC, USA, 1968; pp. 84.
31. United States Bureau of the Census. Vital Statistics and Health and Medical Care. In *Historical Statistics of the United States, Colonial Times to 1970*; Bicentennial Edition, Part 1; U.S. Government Printing Office: Washington, DC, USA, 1975; pp. 44–86.
32. Gordon, J.E.; Hood, R.I. Whooping cough and its epidemiological anomalies. *Am. J. Med. Sci.* **1951**, *222*, 333–361.
33. Cherry, J.D. Pertussis in the Preantibiotic and Prevaccine Era, with Emphasis on Adult Pertussis. *Clin. Infect. Dis.* **1999**, *28* (Suppl. 2), S107–S111.
34. Centers for Disease Control and Prevention. Whooping Cough and the Vaccine (Shot) to Prevent It. In *Information for Parents*; Centers for Disease Control and Prevention: Atlanta, GA, USA, 2015.
35. Quinn, H.E.; Snelling, T.L.; Macartney, K.K.; McIntyre, P.B. Duration of Protection after First Dose of Acellular Pertussis Vaccine in Infants. *Pediatrics* **2014**, *133*, 513–519.
36. Centers for Disease Control and Prevention. *2012 Final Pertussis Surveillance Report*; Final 2012 Reports of Notifiable Diseases; Centers for Disease Control and Prevention: Atlanta, GA, USA, 2013; Volume 62.
37. Centers for Disease Control and Prevention. *2013 Final Pertussis Surveillance Report*; Final 2013 Reports of Notifiable Diseases; Centers for Disease Control and Prevention: Atlanta, GA, USA, 2014; Volume 63.

38. Centers for Disease Control and Prevention. *2014 Final Pertussis Surveillance Report*; Final 2014 Reports of Notifiable Diseases; Centers for Disease Control and Prevention: Atlanta, GA, USA, 2015; Volume 64.
39. United States Bureau of the Census. Table 29—Deaths under 1 year, from selected causes, by age, sex, and race: United States. In *Vital Statistics of the United States 1943 Part 2*; United States Government Printing Office: Washington, DC, USA, 1945; pp. 574–579.
40. United States Bureau of the Census. Table 30—Deaths under 1.year, from selected causes, by age, race, and sex: United States. In *Vital Statistics of the United States 1944 Part 2*; United States Government Printing Office: Washington, DC, USA, 1946; p. 589.
41. United States Public Health Service. Table 26—Deaths under 1 year, from selected causes, by detailed age and race: United States. In *Vital Statistics of the United States 1945 Part 2*; United States Government Printing Office: Washington, DC, USA, 1947; pp. 457–458.
42. Ratner, H.; Cox, H.R.; Greenberg, B.G.; Kleinman, H.; Meier, P. The Present Status of Polio Vaccines. *Ill. Med. J.* **1960**, *118*, 84–93.
43. Wallace, H.M.; Heely, P.; Losty, M.A.; Rich, H. A study of follow-up of poliomyelitis patients. *Am. J. Public Health* **1953**, *43*, 542–553.
44. Ramlow, J.; Alexander, M.; LaPorte, R.; Kaufmann, C.; Kuller, L. Epidemiology of the post-polio syndrome. *Ame. J. Epidemiol*. **1992**, *136*, 769–786.
45. Codd, M.B.; Mulder, D.W.; Kurland, L.T.; Beard, C.M.; O'Fallon, W.M. Poliomyelitis in Rochester, Minnesota, 1935–1955: Epidemiology and Long-term Sequelae: A Preliminary Report. In *Late Effects of Poliomyelitis*; Halstead, L.S., Wiechers, D.D., Eds.; Symposia Foundation: Miami, FL, USA, 1985; pp. 121–134.
46. Brown, G.C.; Lenz, W.R.; Agate, G.H. Laboratory Data on the Detroit Poliomyelitis Epidemic-1958. *J. Am. Med. Assoc.* **1960**, *172*, 807–812.
47. United States Department of Health, Education, and Welfare. Table 54—Deaths from 256 selected causes, by age, race, and sex: United States, 1951. In *Vital Statistics of the United States 1951 Volume 2*; United States Government Printing Office: Washington, DC, USA, 1954; pp. 44–89.
48. Zax, D. The Tonsillectomy: A Massive Pain in the Neck. *Mental Floss Magazine*, 13 March 2011.
49. Anderson, G.W.; Rondeau, J.L. Absence of Tonsils as a Factor in the Development of Bulbar Poliomyelitis. *J. Am. Med. Assoc.* **1954**, *155*, 1123–1130.
50. Centers for Disease Control and Prevention. Poliomyelitis. In *Epidemiology and Prevention of Vaccine-Preventable Diseases*, 11th ed.; Atkinson, W., Wolfe, S., Hamborsky, J., McIntyre, L., Eds; Public Health Foundation: Washington, DC, USA, 2009; pp. 231–244.
51. Nathanson, N.; Kew, O.M. From emergence to eradication: The epidemiology of poliomyelitis deconstructed. *Am. J. Epidemiol.* **2010**, *172*, 1213–1229.
52. Carne, S. Incidence of Tonsillectomy, Circumcision, and Appendicectomy Among R.A.F. Recruits. *Br. Med. J.* **1956**, *2*, 19–23.
53. Fitzmaurice, G. The Odds Ratio: Impact of Study Design. *Nutrition* **2000**, *16*, 1114–1115.
54. Horstmann, D.M. Acute poliomyelitis: Relation of physical activity at the time of onset to the course of the disease. *J. Am. Med. Assoc.* **1950**, *142*, 236–241.
55. Centers for Disease Control and Prevention. Haemophilus influenzae type b. In *Epidemiology and Prevention of Vaccine-Preventable Diseases*, 13th ed.; Hamborsky, J., Kroger, A., Wolfe, S., Eds.; Public Health Foundation: Washington, DC, USA, 2015; pp. 119–134.
56. Dworkin, M.S.; Park, L.; Borchardt, S.M. The Changing Epidemiology of Invasive Haemophilus influenzae Disease, Especially in Persons ≥65 Years Old. *Clin. Infect. Dis.* **2007**, *44*, 810–815.
57. Centers for Disease Control and Prevention. Haemophilus influenzae type b. In *Epidemiology and Prevention of Vaccine-Preventable Diseases*, 11th ed.; Atkinson, W., Wolfe, S., Hamborsky, J., McIntyre, L., Eds.; Public Health Foundation: Washington, DC, USA, 2009; pp. 71–84.
58. Bisgard, K.M.; Kao, A.; Leake, J.; Strebel, P.M.; Perkins, B.A.; Wharton, M. Haemophilus influenzae Invasive Disease in the United States, 1994–1995: Near Disappearance of a Vaccine-Preventable Childhood Disease. *Emerg. Infect. Dis.* **1998**, *4*, 229–237.

59. United Kingdom Department of Health. Haemophilus influenzae type b (Hib). In *Immunisation against Infectious Disease*; The Stationery Office: London, UK, 2006; pp. 127–142.
60. Westerhuis, B.; Bietz, M.G.; Lindemann, J. Acute epiglottitis in adults: An under-recognized and life-threatening condition. *S. D. Med.* **2013**, *66*, 309–313.
61. Silfverdal, S.A.; Bodin, L.; Hugosson, S.; Garpenholt, O.; Werner, B.; Esbjorner, E.; Lindquist, B.; Olce, P. Protective Effect of Breastfeeding on Invasive Haemophilus influenzae Infection: A Case-Control Study in Swedish Preschool Children. *Int. J. Epidemiol.* **1997**, *26*, 443–450.
62. Centers for Disease Control and Prevention. Available online: https://www.cdc.gov/breastfeeding/data/nis_data/index.htm (accessed on 17 December 2016).
63. Reuters. Available online: http://www.reuters.com/article/us-usa-breastfeeding-idUSN0226313220070803 (accessed on 17 December 2016).
64. Centers for Disease Control and Prevention. Background: Adjustments to Reported Cases. In *Viral Hepatitis Surveillance United States*; Centers for Disease Control and Prevention: Atlanta, GA, USA, 2011; pp. 15.
65. Centers for Disease Control and Prevention. A Comprehensive Immunization Strategy to Eliminate Transmission of Hepatitis B Virus Infection in the United States. *Morb. Mortal. Wkly. Rep.* **2006**, *55*, 1–33.
66. Centers for Disease Control and Prevention. Hepatitis B. In *Epidemiology and Prevention of Vaccine-Preventable Diseases*, 13th ed.; Hamborsky, J., Kroger, A., Wolfe, S., Eds.; Public Health Foundation: Washington, DC, USA, 2015; pp. 149–174.
67. Centers for Disease Control and Prevention. Wonder Database Search Query for Deaths from Cirrhosis and Liver Cancer 2010–2014. Available online: https://wonder.cdc.gov/ucd-icd10.html (accessed on 29 October 2016).
68. Alter, M.J.; Hadler, S.C.; Margolis, H.S.; Alexander, W.J.; Hu, P.Y.; Judson, F.N.; Mares, A.; Miller, J.K.; Moyer, L.A. The Changing Epidemiology of Hepatitis B in the United States. *J. Am. Med. Assoc.* **1990**, *263*, 1218–1222.
69. Armstrong, G.L.; Mast, E.E.; Wojczynski, M.; Margolis, H.S. Childhood Hepatitis B Virus Infections in the United States before Hepatitis B Immunization. *Pediatrics* **2001**, *108*, 1123–1128.
70. Centers for Disease Control and Prevention. Hepatitis B. In *Epidemiology and Prevention of Vaccine-Preventable Diseases*, 11th ed.; Atkinson, W., Wolfe, S., Hamborsky, J., McIntyre, L., Eds.; Public Health Foundation: Washington, DC, USA, 2009; pp. 99–122.
71. Centers for Disease Control and Prevention. Varicella. In *Epidemiology and Prevention of Vaccine-Preventable Diseases*, 13th ed.; Hamborsky, J., Kroger, A., Wolfe, S., Eds.; Public Health Foundation: Washington, DC, USA, 2015; pp. 353–376.
72. Centers for Disease Control and Prevention. How Many Cancers Are Linked with HPV Each Year? Available online: https://www.cdc.gov/cancer/hpv/statistics/cases.htm (accessed on 12 April 2017).
73. Centers for Disease Control and Prevention. Wonder Database Search Query for Deaths from Malignant Neoplasm of Cervix Uteri 2011–2014. Available online: https://wonder.cdc.gov/ucd-icd10.html (accessed on 1 November 2016).
74. Blatt, A.J.; Kennedy, R.; Luff, R.D.; Austin, R.M.; Rabin, D.S. Comparison of Cervical Cancer Screening Results Among 256,648 Women in Multiple Clinical Practices. *Cancer Cytopathol.* **2015**, *123*, 282–288.
75. Subramaniam, A.; Fauci, J.M.; Schneider, K.E.; Whitworth, J.M.; Erickson, B.K.; Kim, K.; Huh, W.K. Invasive Cervical Cancer and Screening: What are the Rates of Unscreened and Underscreened Women in the Modern Era? *J. Lower Genit. Tract Dis.* **2011**, *15*, 110–113.
76. American Cancer Society. What are the risk factors for cervical cancer? In *Cervical Cancer Prevention and Early Detection*; American Cancer Society: Atlanta, GA, USA, 2014; pp. 3–7.
77. National Center for Health Statistics. *Chartbook with Special Feature on Adults Aged 55–64: Health Risk Factors. Health, United States, 2014: With Special Feature on Adults Aged 55–64*; National Center for Health Statistics: Hyattsville, MD, USA, 2015; pp. 13–15.
78. Moore, K.A.; Mehta, V. The Growing Epidemic of HPV-Positive Oropharyngeal Carcinoma: A Clinical Review for Primary Care Providers. *J. Am. Board Fam. Med.* **2015**, *28*, 498–503.

79. Centers for Disease Control and Prevention. Wonder Database Search Query for Deaths from Malignant Neoplasms of Lip, Oral Cavity, and Pharynx 2011–2014. Available online: https://wonder.cdc.gov/ucd-icd10.html (accessed on 1 November 2016).
80. Gillison, M.L.; Zhang, Q.; Jordan, R.; Xiao, W.; Westra, W.H.; Trotti, A.; Spencer, S.; Harris, J.; Chung, C.H.; Ang, K.K. Tobacco Smoking and Increased Risk of Death and Progression for Patients With p16-Positive and p16-Negative Oropharyngeal Cancer. *J. Clin. Oncol.* **2012**, *30*, 2102–2111.
81. Gillison, M.L.; D'Souza, G.; Westra, W.; Sugar, E.; Xiao, W.; Begum, S.; Viscidi, R. Distinct Risk Factor Profiles for Human. Papillomavirus Type 16–Positive and Human Papillomavirus Type 16–Negative Head and Neck Cancers. *J. Natl. Cancer Inst.* **2008**, *100*, 407–420.
